# A Challenging Case of a Patient Presenting With Ascites Diagnosed Later As Systemic Sarcoidosis: A Patient Journey Over a Decade

**DOI:** 10.7759/cureus.113149

**Published:** 2026-07-22

**Authors:** Garvit Nama, Ali Elmdaah, Reemah Almadah

**Affiliations:** 1 Internal Medicine, Bristol and Weston University Hospital NHS foundation Trust, Bristol, GBR; 2 Gastroenterology, Aberdeen Royal Infirmary, Aberdeen, GBR; 3 Pharmacology, University of Zawiyah, Alzawiyah, LBY

**Keywords:** angiotensin converting enzyme inhibitors, complex ascites, extra pulmonary manifestations of sarcoidosis, systemic sarcoidosis, systemic steroids

## Abstract

Sarcoidosis is a multi-system granulomatous disease that can affect almost any organ. While pulmonary involvement is common, abdominal sarcoidosis is uncommon and can mimic infections or malignancy. Distinguishing sarcoidosis from abdominal tuberculosis is particularly challenging in patients from endemic regions.

We report a diagnostically complex case of a 38-year-old woman of Bangladeshi origin who initially presented with respiratory and systemic symptoms in 2010. Over more than a decade, she exhibited a broad spectrum of clinical manifestations including bilateral sensorineural hearing loss, chronic anterior uveitis, parotid gland swelling, ascites, omental caking, and abdominal lymphadenopathy. Despite multiple inconclusive investigations and empirical anti-tuberculosis treatments, the final diagnosis of systemic sarcoidosis with abdominal involvement was confirmed via omental biopsy and steroid responsiveness in late 2023. The patient continues on maintenance steroids with no further flare-ups or side effects.

This case highlights that sarcoidosis may present atypically, including with predominant abdominal involvement, and should be considered in patients with chronic multi-system inflammatory disease. The presence of non-caseating granulomas in the context of negative tuberculosis investigations should prompt consideration of sarcoidosis. Misdiagnosis may result in inappropriate treatment and significant delays in care. A favourable response to corticosteroid therapy can support the diagnosis in cases of diagnostic uncertainty. Long-term follow-up remains essential given the relapsing-remitting nature of the disease.

## Introduction

Sarcoidosis is a systemic granulomatous disease of unknown aetiology characterised by the formation of non-caseating granulomas in multiple organs, most commonly the lungs and intrathoracic lymph nodes [1]. The clinical presentation is highly heterogeneous, ranging from asymptomatic cases detected incidentally on imaging to severe multi-organ involvement [2]. While pulmonary involvement occurs in approximately 90% of patients, extra-pulmonary manifestations can affect the skin, heart, nervous system, kidneys, eyes, liver, spleen, musculoskeletal system, haematological system, exocrine and endocrine glands [2,3].

Abdominal involvement in sarcoidosis is uncommon, and when present, it can mimic infections such as tuberculosis or malignancy due to radiological features like ascites, lymphadenopathy, and omental caking [3]. These atypical presentations often lead to diagnostic delays, particularly in populations from regions with a high prevalence of tuberculosis, such as South Asia. Due to these atypical presentations, consideration of common infections such as tuberculosis and leishmaniasis, and lymphoproliferative conditions such as Hodgkin’s lymphoma and peritoneal carcinomatosis becomes important [4,5].

Neurological and auditory involvement, though rare, has also been reported, including sensorineural hearing loss and cranial neuropathies, which may occur in the context of systemic disease [6]. Multi-system involvement, as seen in our patient, exemplifies the challenges in timely recognition and appropriate management of sarcoidosis. Corticosteroids remain the mainstay of treatment for symptomatic sarcoidosis, with response often providing both therapeutic benefit and diagnostic confirmation in ambiguous cases [7]. The rarity of abdominal sarcoidosis and its potential to relapse after initial treatment highlights the importance of long-term follow-up and a multidisciplinary approach.

## Case presentation

A 38-year-old South Asian woman, living in the UK since 2005, first presented to the respiratory clinic in September 2010 with a two-year history of drenching night sweats requiring bed-sheet changes, exertional breathlessness especially in cold air, and intermittent wheeze without cough, sputum or haemoptysis. She also reported progressive bilateral swelling and stiffness of the small joints of the hands, affecting morning function and daily tasks, together with a gradually enlarging right parotid swelling. She denied weight loss, visual disturbance, neurological symptoms or prior tuberculosis (TB). Her background included beta-thalassaemia trait and long-term betel nut chewing. At this point, she has had 2 years of sudden-onset bilateral sensorineural hearing loss (SNHL), for which an MRI Brain was reported to be normal in 2008. There was no significant family history of TB or hereditary or autoimmune conditions. She was a mother of six children and has been a housewife. Examination revealed unilateral parotid enlargement and bi-basal fine inspiratory crackles, with no lymphadenopathy or organomegaly.

**Figure 1:**
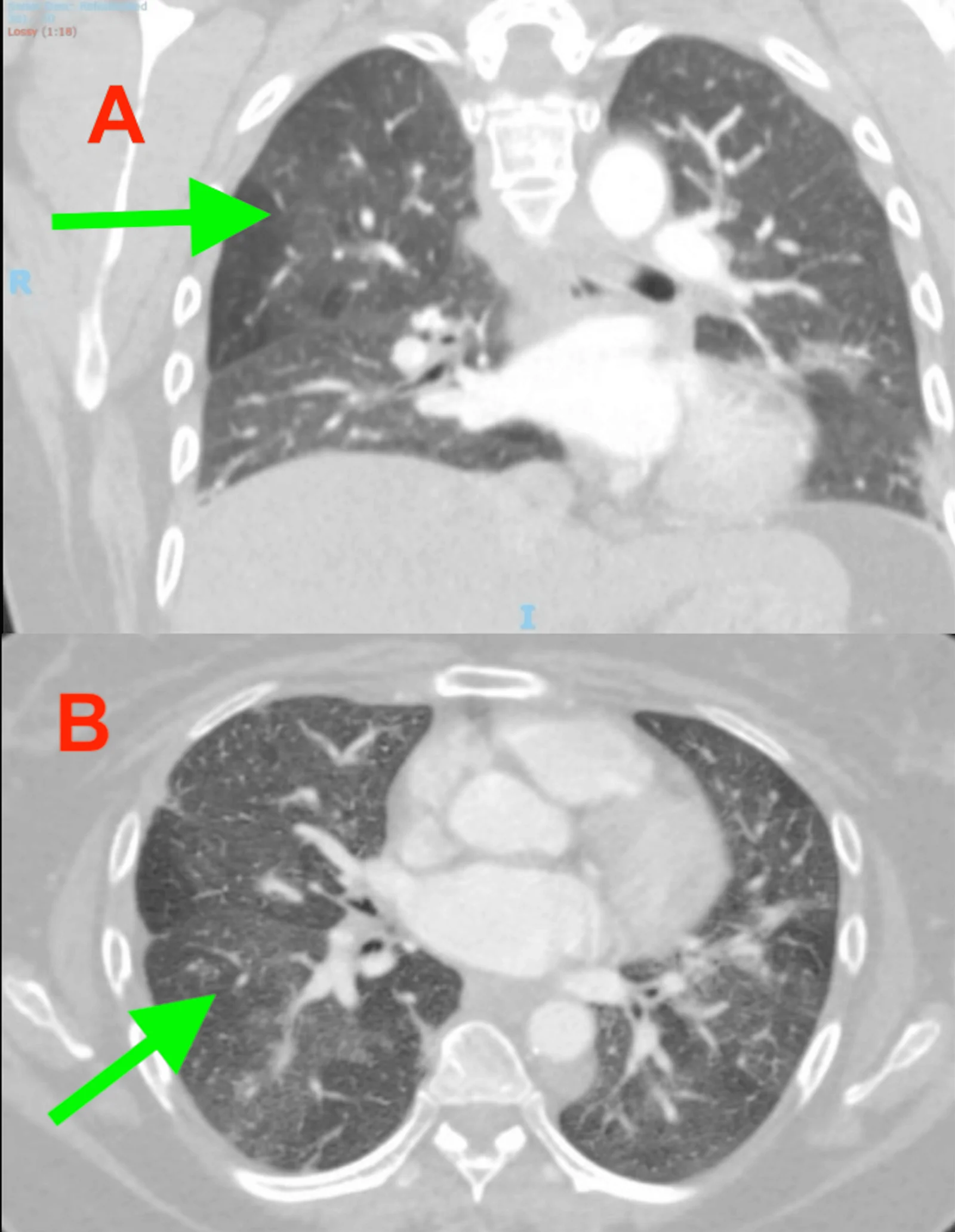
CT Chest (10/2010) - coronal view (A) and transverse view (B) showing bilateral ground-glass opacification of the lung fields (green arrows)

**Figure 2:**
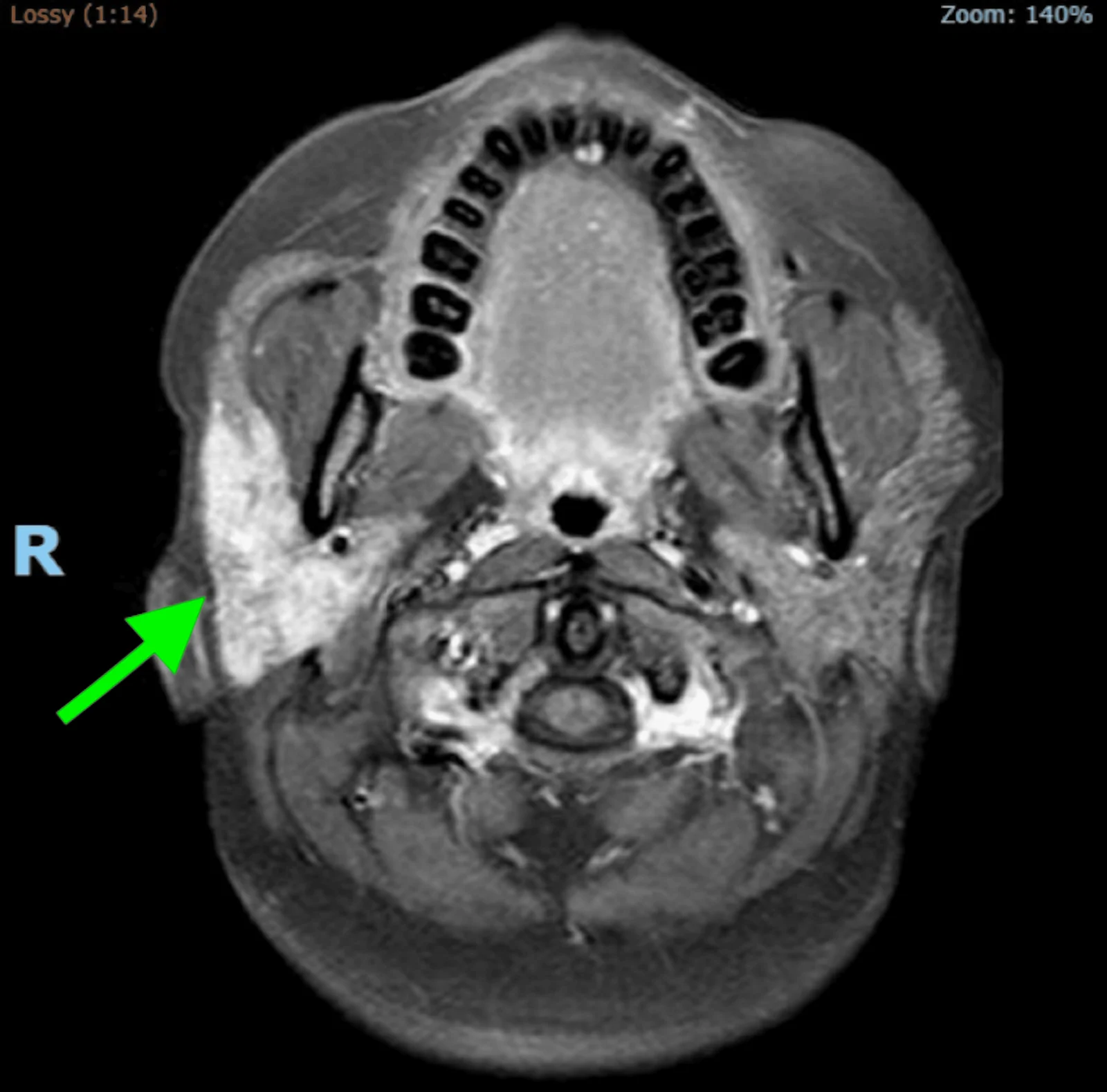
MRI Neck (05/2010) - T1 gadolinium (GAD) spectral inversion recovery (SPUR) transverse section showing edema and marked enhancement of the right parotid gland, suggesting chronic suppurative or non-suppurative parotitis

Initial investigations included CT chest (Figure 1) demonstrating faint ground-glass attenuation and small mediastinal lymph nodes, felt potentially artifactual. MRI Head and neck (Figure 2) revealed right-sided chronic parotitis (suppurative or non-suppurative), and further biopsy revealed an epithelioid granuloma. Bronchoscopy was normal, with acid-fast bacilli (AFB)-negative washings (Table 1). Angiotensin-converting enzyme (ACE) levels were borderline, and ESR was mildly elevated at this point (Table 2). Autoimmune studies and rheumatoid serology were negative, while serum electrophoresis showed polyclonal hypergammaglobulinaemia (raised IgG and IgA) (Table 3). T-spot (Tuberculin skin spot) testing fluctuated between borderline and negative (Table 2). Considering her ethnicity, TB was a key differential, though early features suggested possible sarcoidosis.

**Table 1:** Respiratory investigations over the course of 13 years, including bronchial washings and biopsy, along with CT Chest findings AFB, acid-fast bacilli (stain); TB, tuberculosis; NSIP, nonspecific interstitial pneumonia

Date	Investigation	Findings	Comments
09/2010	Bronchial washings	AFB negative	TB less likely
10/2010	Bronchial biopsy	Chronic and acute inflammation, no granuloma, dysplasia or malignancy, AFB stain negative for TB	TB not likely
10/2010	CT Chest	Minor lymphadenopathy (10 mm posterior mediastinal lymph node with few lymph nodes noted in axilla) but not reaching up to threshold, ground-glass opacification - likely motion artefact	No sarcoidosis evidence
01/2017	CT Chest	Bilateral mosaic ground-glass opacity with subpleuritic fibrotic changes are concerning for possible interstitial lung disease (NSIP pattern). Multiple subcentimetre lymph nodes are likely reactive in nature.	Possible NSIP
07/2023	CT Chest	Florid mosaic attenuation with middle lobe bronchial traction bronchiectasis and areas of subpleural cystic changes in the chest that remain relatively stable. Posterior mediastinal adenopathy has reduced in size	Adenopathy reduced

**Table 2:** Lab values for ACE, T-spot, and ESR trends over 13 years ACE, angiotensin-converting enzyme; T-spot, Tuberculin skin spot test; ESR, erythrocyte sedimentation rate

Date	Test	Value/Result	Reference	Comments/Interpretation
09/2010	ACE	47 U/L	16-52 U/L	Borderline
09/2014	ACE	30 U/L	16-52 U/L	Normal
03/2017	ACE	56 U/L	16-52 U/L	Borderline
04/2023	ACE	88 U/L	16-52 U/L	Elevated
2023 (post steroids)	ACE	23-39 U/L	16-52 U/L	Decreased with therapy
09/2010	T-spot	Borderline	Negative	Inconclusive for latent/active TB
10/2010	T-spot	Non-reactive	Negative	Unlikely to be latent/active TB
10/2017	T-spot	Non-reactive	Negative	Unlikely to be latent/active TB
2008–2019	ESR	22-44 mm/hr	1-12 mm/hr	Mild to moderate elevation
09/2022	ESR	84 mm/hr	1-12 mm/hr	Significant inflammation
04/2023	ESR	105 mm/hr	1-12 mm/hr	Peak value before steroid initiation
05/2023	ESR	47 → 18 mm/hr	1-12 mm/hr	Improvement after steroids
08/2023	ESR	5 mm/hr	1-12 mm/hr	On steroid maintenance

**Table 3:** Lab values for ANA, RF, Anti-CCP, HEp-2, ANCA, MPO, PR3, IgG, IgM, IgA, and IgG4 trend over 13 years. ANA, antinuclear antibody; ANCA, antineutrophil cytoplasmic antibody; Anti-CCP, anti-cyclic citrullinated peptide antibodies; HEp-2, human epithelial type 2; IgA, immunoglobulin A; IgG, immunoglobulin G; IgG4, immunoglobulin G4 subtype; IgM, immunoglobulin M; MPO, myeloperoxidase; PR3, proteinase 3; RF, rheumatoid factor.

Date	Test	Value/Result	Reference	Comments/Interpretation
09/2010	ANA	Negative	Negative	Unlikely to be an autoimmune process
09/2010	Rheumatoid screen	RF (9 IU/ml), Anti-CCP (3 U/ml)	RF (<20 IU/ml), Anti-CCP (<9 U/ml)	Negative rheumatoid arthritis screen
05/2023	ANA	Positive (HEp-2)	Negative	Autoimmune process indicator
08/2023	Complement C3/C4	C3 (1.5 g/L), C4 (0.3 g/L)	C3 (0.9-1.8 g/L), C4 (0.1-0.4 g/L)	Normal
05/2023	ANCA (MPO, PR3)	Negative	Negative	Normal
09/2010	IgG	22 g/L	7-16 g/L	Elevated
09/2010	IgA	4.5 g/L	0.7-4 g/L	Mildly elevated
09/2010	IgM	2.2 g/L	0.4-2.5 g/L	Normal
08/2022 to 08/2023	IgG	25 → 13.9 g/L	7-16 g/L	Decreasing trend
08/2022 to 08/2023	IgA	5.1 → 3 g/L	0.7-4 g/L	Slight decline
08/2022 to 08/2023	IgM	0.71 → 1.07 g/L	0.4-2.5 g/L	Slight increase
08/2023	IgG4	0.44 g/L	<1 g/L	Normal

Over the subsequent months, she developed new multi-system involvement. By late 2010, she had suffered profound bilateral sensorineural hearing loss for 2 years, progressing to complete deafness, requiring cochlear implantation, accompanied by worsening hand stiffness and intermittent right eye erythema. Repeat CT chest again showed subtle ground-glass change without clear evidence of sarcoidosis or interstitial lung disease. Repeat bronchoscopy remained non-diagnostic, and repeat T-spot was negative (Table 1 and 2).

She remained relatively stable until November 2012, when she presented acutely with abdominal pain, fever, worsening night sweats, dry cough, and significant abdominal distension. Computed Tomography of the Chest, Abdomen, and Pelvis (CT CAP) with contrast showed large-volume ascites, omental caking, retroperitoneal and pelvic lymphadenopathy, and prominent mediastinal lymph nodes. Peritoneal fluid cytology demonstrated a lymphocyte-rich exudate with no malignancy. Omental biopsy showed multiple epithelioid granulomas with Langhans giant cells, and stains for AFB and fungi were negative (Table 4). Despite microbiological negativity, the histological impression and clinical severity led to empirical treatment for presumed peritoneal TB with a six-month anti-TB regimen. Symptoms partially improved with the initial regimen.

**Table 4:** List of major procedures and imaging for the last 13 years AAFB, acid-alcohol-fast bacilli; OGD, esophagogastroduodenoscopy; PCR, polymerase chain reaction; ZN, Ziehl-Neelsen (stain); CT CAP: computed tomography of the chest, abdomen, and pelvis; TB, tuberculosis; US, ultrasonography

Date	Procedure/Imaging	Findings	Comments/Indication
10/2012	CT CAP with contrast	Ascites with omental caking, bulky cervix. Enlarged para-aortic and bilateral iliac lymph nodes and prominent mediastinal lymph nodes. Appearances are most likely due to an underlying malignancy; however, tuberculosis should be considered	Suspicion of malignancy vs TB
11/2012	Peritoneal Fluid	No malignant cells seen. Mesothelial cells with background of numerous lymphoid cells predominantly including lymphocytes and occasional polymorph neutrophils	Inflammatory process
11/2012	Omental & Peritoneal Biopsy	Granulomatous inflammation. Multiple epithelioid cell granulomas, with multinucleate giant cells, Langhans type and foreign body type, no evidence of malignancy, and stains for AAFB and fungi are negative	Morphology supports TB
12/2013	OGD	Normal	Due to abdominal pain and gastric reflux
01/2014	Duodenal Biopsy	Within normal morphological limits (Marsh type 0). Normal villi with no increase in the intraepithelial lymphocytes or crypt mitoses with no parasites	Rules out coeliac
09/2014	US Abdomen	Fatty liver with no ascites	Symptom-driven (abdominal distension)
12/2016	CT CAP with contrast	Mild progression of disease with mild progression in associated adenopathy since the last scan in 10/2012. Increase in left retrocrural node and right iliac node in specific.	Mild disease progression
11/2017	Oral Biopsy	Benign keratosis	Bilateral leukoplakia, betel nut chewer
05/2018	OGD	Small hiatus hernia with Barrett’s	Due to dysphagia
05/2018	Lower oesophageal and gastric antrum biopsy	The former is within normal limits, and the latter shows reactive gastritis	Due to dysphagia
08/2022	CT CAP with contrast	Distended abdomen is due to large volume ascites? Active TB and progressive adenopathy, particularly in the pelvis. Increase in size of right iliac node, with persistent retroperitoneal, common iliac and external iliac adenopathy	Suspect TB again
09/2022	Ascitic Fluid Cytology and culture	Lymphocyte-rich fluid, no malignant cells; cultures, smear microscopy, and PCR were negative for TB	Inflammatory condition, TB unlikely
09/2022	Right external Iliac lymph node core biopsy	Granulomatous inflammation, ZN stain was negative, but unfortunately, the sample was not sent for TB culture	Inflammatory condition
07/2023	CT CAP with contrast	Overall disease improvement. Florid mosaic attenuation with middle lobe bronchial traction bronchiectasis and areas of subpleural cystic changes in the chest that remain relatively stable. Posterior mediastinal adenopathy has reduced in size, and previous retroperitoneal and pelvic adenopathy has completely resolved. Complete resolution of ascites.	Post-steroid treatment

Between 2013 and 2016, she experienced episodic joint stiffness, intermittent cough, and fluctuating night sweats, with long gaps in follow-up. A cervical biopsy performed in 2015 demonstrated cervical intraepithelial neoplasia grade 1 (CIN1) with focal areas of cervical intraepithelial neoplasia grade 2 (CIN2), consistent with a florid human papillomavirus (HPV)-related epithelial response. By 2016, CT imaging showed mild progression of retroperitoneal and iliac lymphadenopathy but no definite malignancy or TB (Table 4). 

**Figure 3:**
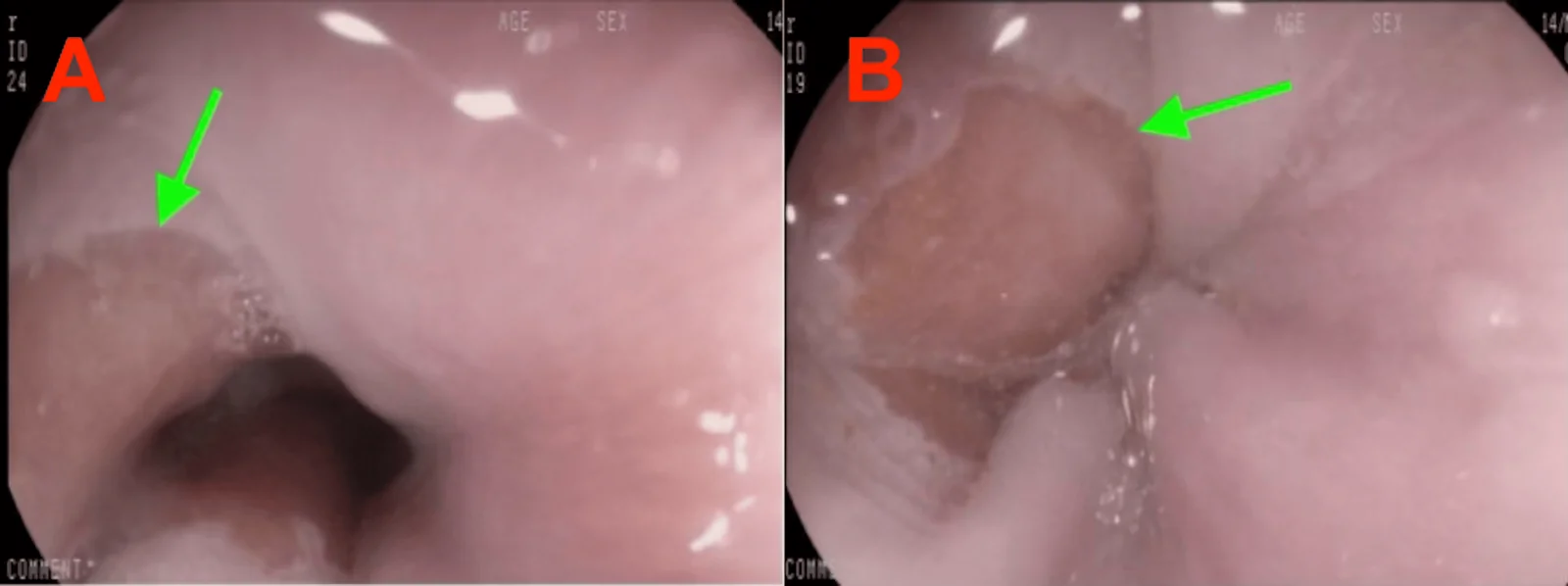
Oesophago-gastro-duodenoscopy (A and B) (05/2018) showing extended Z-line (Green arrows) into the lower oesophagus, consistent with Barrett’s oesophagus

In 2017, she re-presented with recurrent abdominal distension, fevers, and lymphadenopathy. CT chest showed bilateral mosaic ground-glass opacities and early fibrotic subpleural change, raising concern for nonspecific interstitial pneumonia, though respiratory review suggested possible pulmonary sarcoidosis (Table 1). Repeat T-spot testing remained negative (Table 2). She has also undergone an oesophago-gastro-duodenoscopy in mid-2018 due to dysphagia, which showed a small hiatus hernia with Barrett’s oesophagus, and the gastric antral biopsy revealed reactive gastritis (Figure 3, Table 4)

Over the next several years (2018-2021) she reported intermittent joint pain, mild shortness of breath on exertion, occasional cough, and fluctuating fatigue, but remained clinically stable from a respiratory standpoint. No sustained follow-up occurred with rheumatology despite referrals.

**Figure 4:**
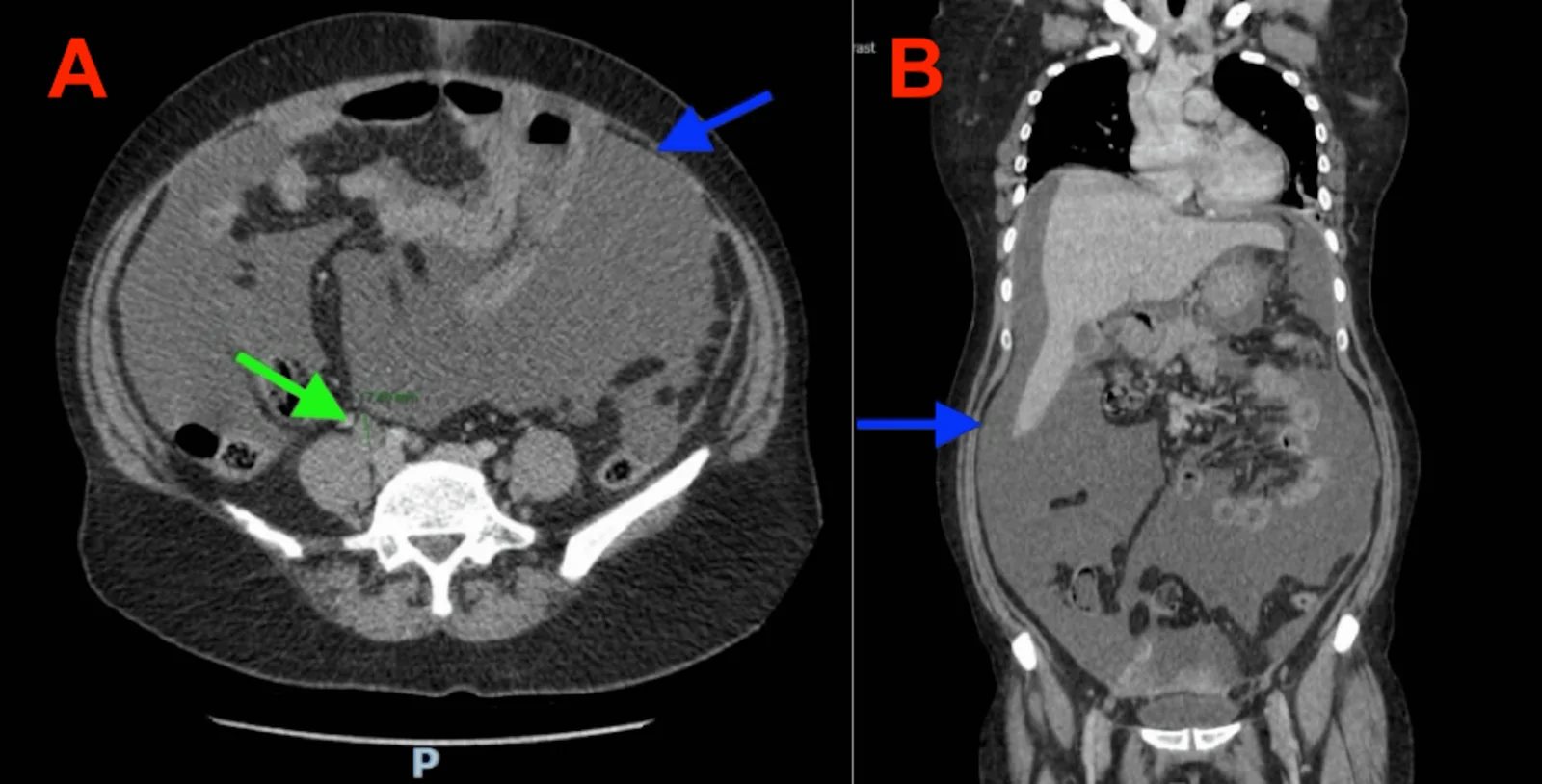
CT Chest, abdomen and pelvis with contrast (08/2022) - transverse (A) and coronal (B) sections, showing significant ascites with omental caking (blue arrows), and enlarged retroperitoneal lymph nodes (green arrow)

In 2022 her symptoms again escalated. She developed gradual yet significant abdominal distension from April 2022 onwards, with associated constipation, low back pain, worsening fatigue and recurrent night sweats. CT chest abdomen pelvis (CAP) with contrast in August 2022 demonstrated large-volume ascites, marked peritoneal nodularity, progressive pelvic and retroperitoneal lymphadenopathy, and stable but abnormal basal lung changes (Figure 4). Ascitic fluid was repeatedly negative for malignant cells and TB smear microscopy (TB smear), polymerase chain reaction (PCR), and mycobacterial cultures. A core biopsy of a right external iliac node showed granulomatous inflammation with negative Ziehl-Neelsen (ZN) stain; unfortunately, no sample was sent for TB culture for the iliac nodes at this time (Table 4).

In January 2023, she developed new bilateral pre- and post-auricular swellings (4-5 cm on the right, around 2 cm on the left). Laboratory tests showed persistently raised ESR (84-105 mm/hr), hypergammaglobulinaemia (IgG up to 27 g/L), and an elevated ACE level (88 U/L). Rheumatoid factor was weakly positive, anti-nuclear antibody (ANA) weakly positive, and IgG4 was normal. T-spot remained negative (Table 2 and Table 3). The recurring pattern of granulomatous inflammation affecting parotids, omentum, lymph nodes, lungs and consistent microbiological negativity began to strongly favour systemic sarcoidosis rather than TB.

At the Respiratory multidisciplinary team (MDT) review in April 2023, the decade‑long history of multi‑system granulomas, fluctuating constitutional symptoms, polyclonal hypergammaglobulinaemia, and inadequate response to previous anti‑tuberculosis (TB) treatment was considered more consistent with sarcoidosis than TB. As a result, anti‑TB therapy was discontinued in May 2023, and the patient was commenced on prednisolone at a loading dose of 30 mg.

**Figure 5:**
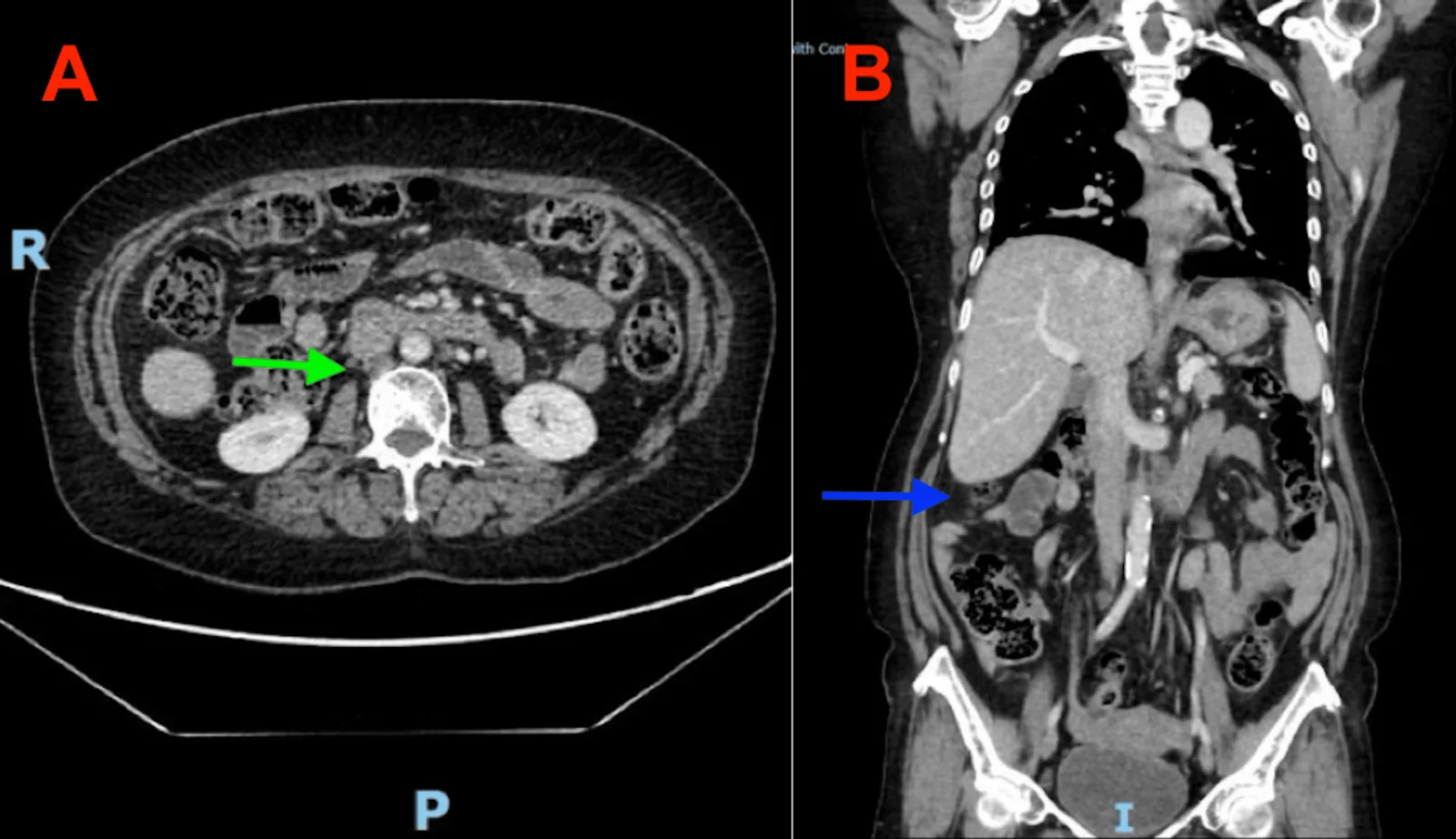
CT Chest, abdomen and pelvis with contrast (07/2023) - transverse (A) and coronal (B) sections, showing marked improvement in the retroperitoneal lymphadenopathy (green arrow) and complete resolution of ascites (blue arrow)

She exhibited marked improvement within weeks. Night sweats, abdominal distension and systemic symptoms resolved entirely. ESR dropped from 105 to 18 and later to 5 mm/hr, and her ACE normalised (Table 2). By July 2023, CT CAP with contrast showed complete resolution of ascites and major reduction of pelvic and retroperitoneal lymphadenopathy (Figure 5). Mediastinal nodes decreased, and ground-glass changes in the lungs remained stable (Table 1).

By late 2023, she continued to remain clinically stable on a tapering steroid regimen, with only mild bilateral facial swelling and stable pulmonary changes. Her abdominal symptoms did not recur, and she felt significantly better overall. She is now 53 years old, currently in remission on maintenance prednisolone 4 mg daily, along with proton pump inhibitors (PPIs) and calcium and vitamin D tablets, with ongoing respiratory and rheumatology follow-up.

## Discussion

Sarcoidosis is a systemic granulomatous disease of unknown aetiology that most commonly affects the lungs and intrathoracic lymph nodes. While it can involve virtually any organ, abdominal sarcoidosis remains rare and is often misdiagnosed as malignancy or abdominal tuberculosis, particularly in populations from endemic TB regions like South Asia.

Our patient’s case highlights several unique diagnostic and management challenges, as listed below.

Atypical organ involvement and delayed diagnosis

While pulmonary involvement is seen in over 90% of sarcoidosis cases, intra-abdominal manifestations such as omental caking, ascites, and mesenteric lymphadenopathy are uncommon [8]. A review by Warshauer and Lee noted that abdominal sarcoidosis can mimic peritoneal carcinomatosis or TB due to similar radiologic features like ascites and lymphadenopathy [3,9]. In this case, repeated TB investigations were negative over the years, and granulomatous inflammation was misattributed to TB, delaying proper immunosuppressive therapy.

Diagnostic complexity

Diagnosing sarcoidosis remains a clinical challenge due to its non-specific presentation [1]. The American Thoracic Society (ATS) diagnostic criteria require: clinical/radiologic presentation consistent with sarcoidosis, histologic evidence of non-caseating granulomas, and exclusion of other granulomatous diseases like tuberculosis or fungal infections [4].

Our patient met all three criteria, with non-caseating granulomas in the parotid and omental biopsies, radiologic lung and abdominal involvement, and persistently negative TB tests and cultures. Multiple elevated ESR readings, elevated ACE levels at the time of ascites and retroperitoneal lymphadenopathy, and polyclonal hypergammaglobulinemia supported chronic inflammation [2].

Multi-system involvement and consideration of important differentials

The case is further complicated by involvement of the auditory system (bilateral SNHL), ocular system (anterior uveitis), salivary glands (bilateral parotid swelling), and gastrointestinal tract, all of which are recognised but uncommon manifestations of systemic sarcoidosis [10]. Sarcoidosis-induced SNHL is rare but increasingly reported in the literature, often in association with central nervous system involvement [11]. In our patient, however, it appeared to form part of a broader chronic inflammatory process affecting multiple organ systems.

Tuberculosis remained a key differential diagnosis given the patient’s ethnic background, abdominal imaging findings, intermittent partial response to anti-TB therapy, and granulomatous inflammation on omental biopsy. In the absence of clear epidemiological information and clinical context, distinguishing between sarcoidosis and tuberculosis can be challenging. Although T-spot testing, PCR, and culture results help guide decision-making, they must ultimately be interpreted alongside the overall clinical picture [12].

IgG4-related disease was also considered due to the overlapping features (parotid swelling, uveitis, SNHL), but this was deemed unlikely in view of the patient’s low IgG4 levels, the histopathological findings, and the favourable response to corticosteroid therapy [13].

Therapeutic response

The dramatic and sustained response to oral corticosteroids confirms the inflammatory nature of the disease. The European Respiratory Society and ATS recommend systemic corticosteroids as the first-line treatment for symptomatic or extra-pulmonary sarcoidosis, as there is no cure for sarcoidosis [14,15]. Steroid-sparing agents such as methotrexate, azathioprine, or mycophenolate are used for chronic or refractory disease, or in cases of inability to taper steroids. Biologics, particularly tumour necrosis factor alpha (TNF-α) inhibitors like infliximab and adalimumab, are reserved for severe or resistant cases [15]. Ultimately, the holistic management of sarcoidosis requires a three-pronged approach, i.e., treatment of symptomatic granulomatous inflammation, moderating immunosuppressive toxicities, and assessing co-morbidities.

When she started on a loading dose of steroids, her ESR normalised within months, ascites resolved, and systemic symptoms improved significantly. She was able to be weaned off to a daily maintenance dose of 5 mg and remains well on this. 

## Conclusions

This case highlights the need to actively consider sarcoidosis in patients with multi-organ granulomatous inflammation, even when the presentation is uncommon. Predominant abdominal involvement with ascites and associated sensorineural hearing loss closely mimicked peritoneal tuberculosis, resulting in repeated courses of unnecessary anti-tuberculous therapy. Persistent symptoms despite treatment, repeatedly negative tuberculosis investigations, and widespread non-caseating granulomas ultimately favoured sarcoidosis.

The diagnosis was supported by histological findings and a clear clinical response to corticosteroids, with inflammatory markers and polyclonal hypergammaglobulinaemia reflecting disease activity. This case emphasises the importance of timely reassessment, repeat histology, and multidisciplinary input when disease behaviour is inconsistent with the presumed diagnosis. Ongoing follow-up remains essential given the relapsing nature observed in this patient.
